# Benchmarking machine learning models for late-onset alzheimer’s disease prediction from genomic data

**DOI:** 10.1186/s12859-019-3158-x

**Published:** 2019-12-16

**Authors:** Javier De Velasco Oriol, Edgar E. Vallejo, Karol Estrada, José Gerardo Taméz Peña, The Alzheimer’s Disease Neuroimaging Initiative

**Affiliations:** 1 Department of Bioinformatics, Escuela de Medicina y Ciencias de la Salud, Tecnologico de Monterrey, Monterrey, 64710 Mexico; 2 Graduate Professional Studies, Brandeis University, Waltham, 02453 MA USA

**Keywords:** Alzheimer’s disease, Machine learning, Benchmarking, Genome-wide association studies

## Abstract

**Background:**

Late-Onset Alzheimer’s Disease (LOAD) is a leading form of dementia. There is no effective cure for LOAD, leaving the treatment efforts to depend on preventive cognitive therapies, which stand to benefit from the timely estimation of the risk of developing the disease. Fortunately, a growing number of Machine Learning methods that are well positioned to address this challenge are becoming available.

**Results:**

We conducted systematic comparisons of representative Machine Learning models for predicting LOAD from genetic variation data provided by the Alzheimer’s Disease Neuroimaging Initiative (ADNI) cohort. Our experimental results demonstrate that the classification performance of the best models tested yielded ∼72% of area under the ROC curve.

**Conclusions:**

Machine learning models are promising alternatives for estimating the genetic risk of LOAD. Systematic machine learning model selection also provides the opportunity to identify new genetic markers potentially associated with the disease.

## Background

Alzheimer Disease (AD) is a neurodegenerative disorder that gradually destroys brain function. It is characterized by the loss of cognitive abilities such as memory, reasoning, language, and behavior. The disease leads to dementia and ultimately to death. AD is the most common form of dementia (60% – 80% cases) and occurs more often in people aged 65 and older[1]. Age is not the only risk factor for developing AD, it has been observed that there are specific inherited genetic traits that increase the risk of Early-Onset AD (EOAD) at an early age (<60). Apart from the age differences, the clinical presentation of EOAD is very similar to the presentation of late-onset AD (LOAD) and many aspects of the disease overlap with normal again in many clinical and pathological aspects. The EOAD by family inheritance is characterized by genetic mutations in the APP, PSEN1, and PSEN2, related to amyloids but only accounts for 5% of total AD [2].

The high prevalence of LOAD among the elderly is caused by the increasing life expectancy coupled with the lack of an effective treatment to either stop the advance of the sickness or reverse the damage caused by it; and up to this date, there are only two FDA-approved drugs to treat AD cognitive symptoms. An estimate from Ballard [3] shows that Alzheimer’s Disease affects between 4 and 6 percent of the population around 65 years old, that the incidence of the disease doubles every five years after 65 years of age, and by age of 85 between 30%-50% is affected by some form of AD. Therefore, there are a lot of efforts aimed at developing effective AD therapies, and it is expected that preventive ones have a greater impact before the development of the disease [4]. To apply these preventive treatments, a key component is detecting those individuals at risk at an early stage of the disease. There are multiple existing methods such as cognitive tests, magnetic resonance imaging (MRI), positron emission tomography (PET) images, cerebrospinal and blood biomarkers that can determine the development of AD [5]. But these methods do not detect the formation or propensity of the disease at a sufficiently early stage to be highly effective. Additionally, pathological postmortem examination is required for confirmatory diagnosis [6]. To complicate matters further, these biomarkers and MRI features develop in a correlated manner with the development of the disease and are at their most usefulness for prediction when the disease has progressed to the final stages.

A promising method for improving the prediction of LOAD is through the study of risk factors, and genetic testing has become an important source of information that can profile the genetic component of LOAD risk. One specific case is the gene Apolipoprotein E(APOE) and its different alleles, which have been implicated as the largest genetic risk factors for LOAD. Late-Onset Alzheimer’s Disease is a complex multifactorial disease; thus, the APOE variants do not give a definite prediction of the disease by themselves.

Multiple other genes such as CLU, PICALM, CR1 [7] have been shown to be statistically correlated and biochemically plausible. These common variants found using multiple genome-wide association studies (GWAS) have been shown to explain only 33% of the phenotypic variance of LOAD, while the expected heritability component of LOAD is around 79%, thus leaving over 40% unexplained [8]. LOAD is expected to have a known genetic component, a missing (so far) genetic component, and multiple environmental factors that contribute to the complexity of the disease [9].

The complexity of LOAD can be studied using modern machine learning (ML) strategies that leverage well-planned AD studies. With the aim to discern and discover the multiple factors that affect the onset of AD, the Alzheimer’s Disease Neuroimaging Initiative (ADNI) launched a longitudinal study to: “develop clinical, imaging, genetic, and biochemical biomarkers for the early detection and tracking of Alzheimer’s disease (AD)”. The first goal of the study is: “To detect AD at the earliest possible stage (pre-dementia) and identify ways to track the disease’s progression with biomarkers” [10]. Therefore, ADNI is a well-planned study that produces the required data to be data mined by ML. There have been several machine learning strategies that have been used to explore early stages of AD [11–13]. Most of the ML approaches are based on exploring univariate associations with MCI to AD conversions [13], and some efforts have been made in building predictive multivariate models based on merging clinical, MRI, laboratory and PET imaging [14]. These efforts have been very successful, and there are several alternatives to predict the early stages of LOAD [15]. On the other hand, similar ML approaches can be used to predict AD risk based on gene variants; but most of the efforts have been constrained to the use of advanced statistical approaches [16]. To fully explore the potential of gene biomarkers in the prediction of LOAD, multivariate ML is required. The number of approaches to be explored is very large, and their validation requires complex exploration of prediction performance and evaluation of the internal structure, i.e., what are the Single Nucleotide Polymorphisms (SNP) involved in the successful prediction of LOAD? Hence, the aim of this work was to explore the performance of genetic-based ML multivariate strategies in predicting LOAD and to describe the main genetic features associated with the risk of developing LOAD.

To achieve this goal, we used the benchmark tool implemented in FRESA.CAD (Feature Selection Algorithms for Computer Aided Diagnosis) [17, 18]. The benchmark tool evaluates statistical feature selection methods, wrapper/filter ML methods, and the ensemble of models in a coherent cross-validation and repetition method yielding a high degree of statistical confidence of the test performance. FRESA.CAD additionally has the advantage of returning the features most selected across the models and can extrapolate to a valid analysis of the gene variants which allows a more direct interpretation. We propose the hypothesis that the FRESA.CAD Benchmarking tool can achieve high predictive results by comparing and analyzing multiple Machine Learning models applied to predict the genetic risk a person has of developing Alzheimer’s Disease from genetic information only. We expect these models to explain more of the missing heritability than simpler models as the methods can represent nonlinearities from gene interactions and use a broader amount of SNPs in contrast to single markers from GWAS.

## Results

**Fig. 1:**
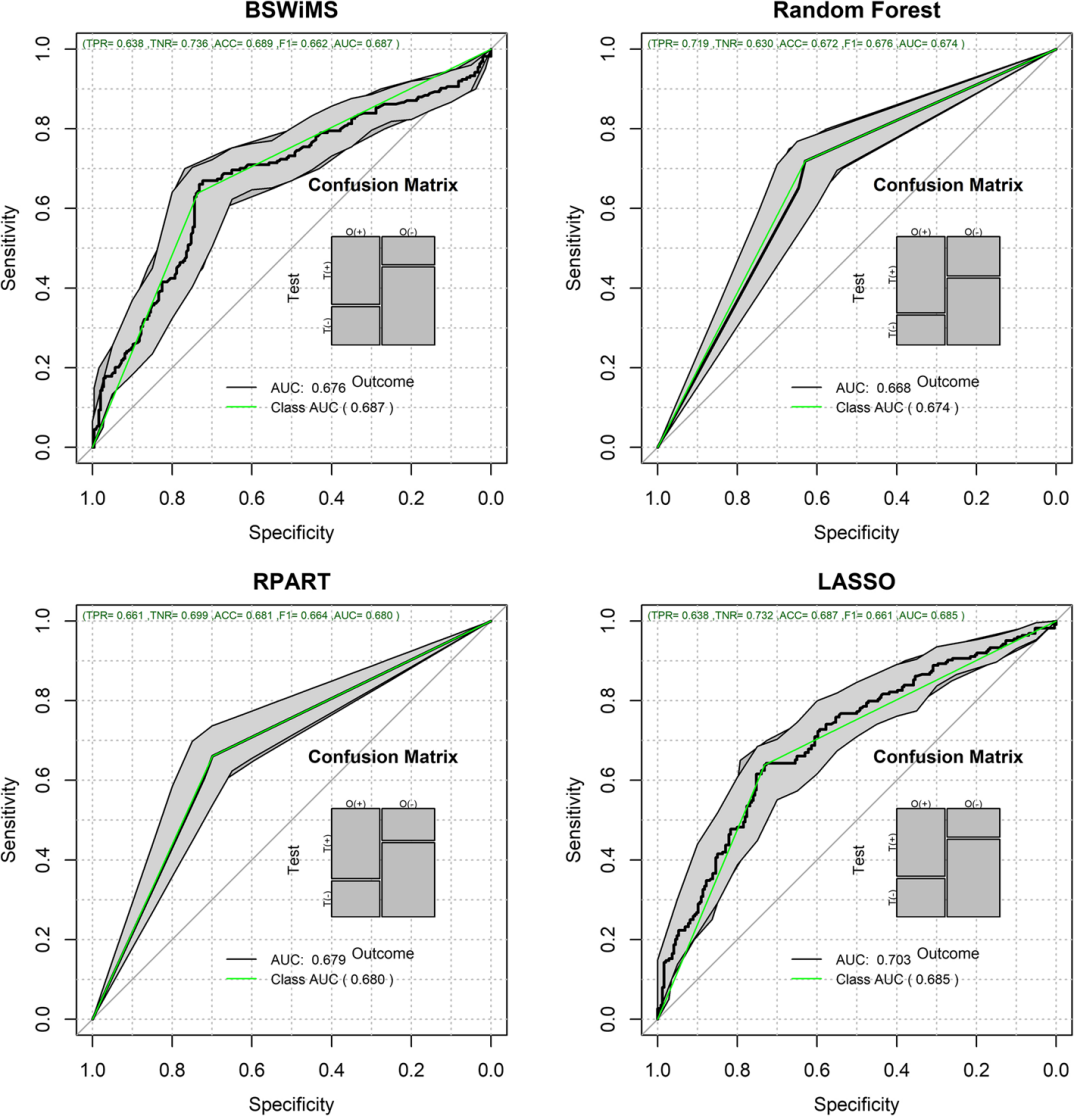
ROC Curves for the FRESA.CAD Benchmarking Classifiers ROC Curves obtained using BSWiMS, Random Forest, RPART and LASSO of the FRESA.CAD Benchmarking with the ADNI-Discovery dataset for the Cross-Validation and the top 2,500 SNPs as inputs

**Fig. 2:**
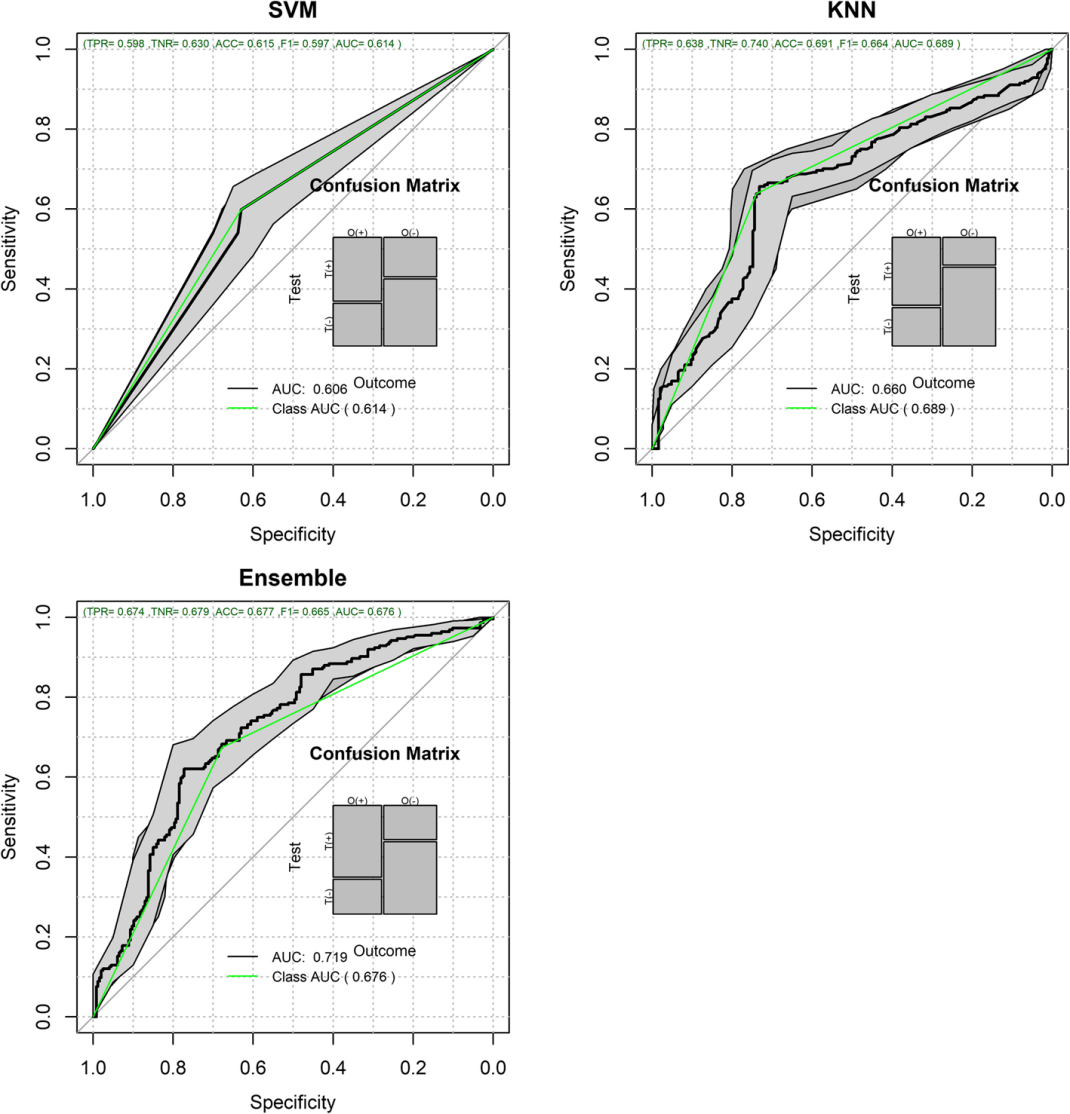
ROC Curves for the FRESA.CAD Benchmarking Classifiers (Continued) ROC Curves obtained using SVM, KNN and the Ensemble of the FRESA.CAD Benchmarking with the ADNI-Discovery dataset for the Cross-Validation and the top 2,500 SNPs as inputs

**Fig. 3:**
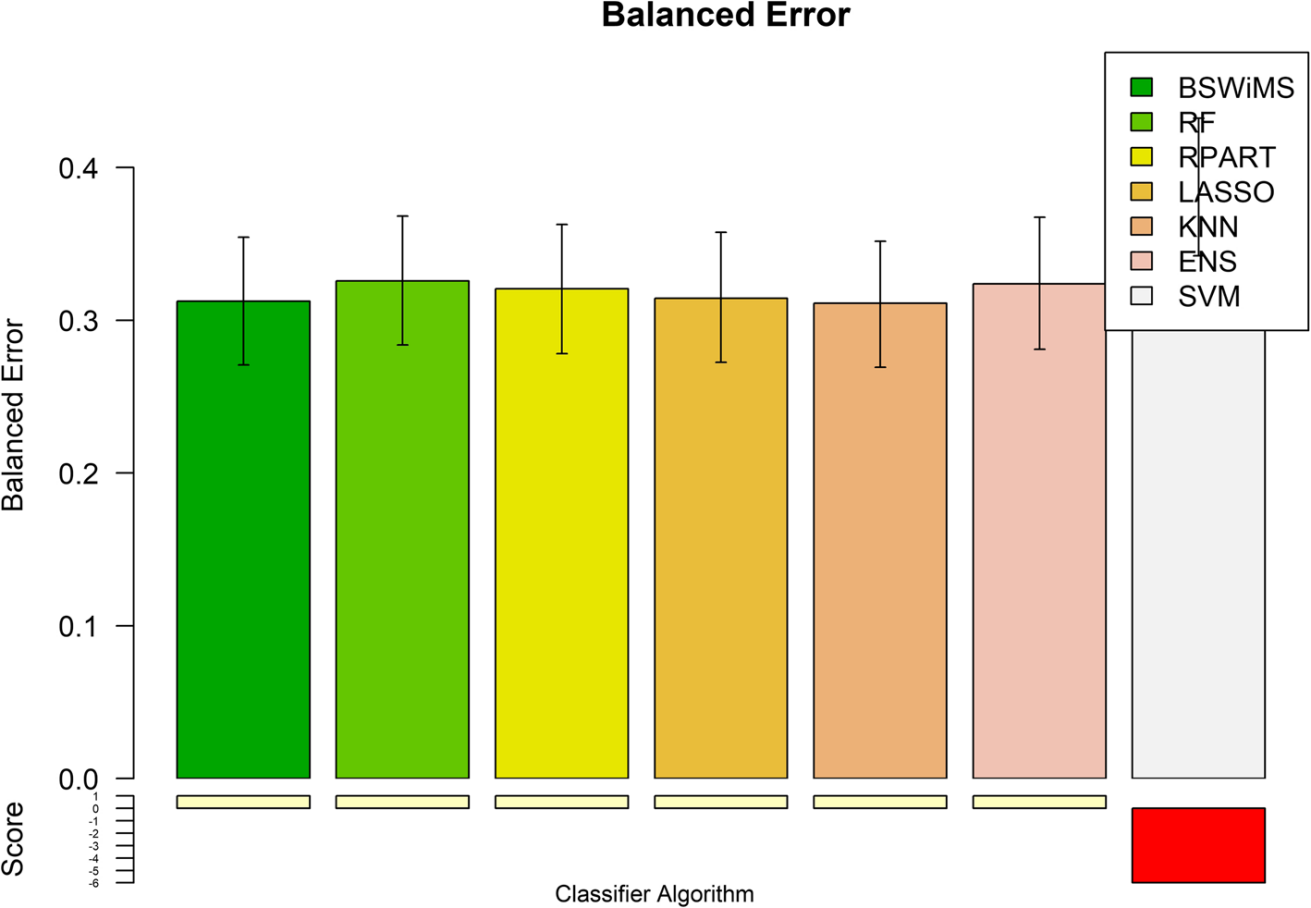
Balanced Error of the FRESA.CAD Benchmark classifiers Comparison of the Balanced Error obtained using the different classification methods of the FRESA.CAD Benchmarking with the ADNI-Discovery dataset for the Cross-validation and using the top 2500 SNPs as input

**Fig. 4:**
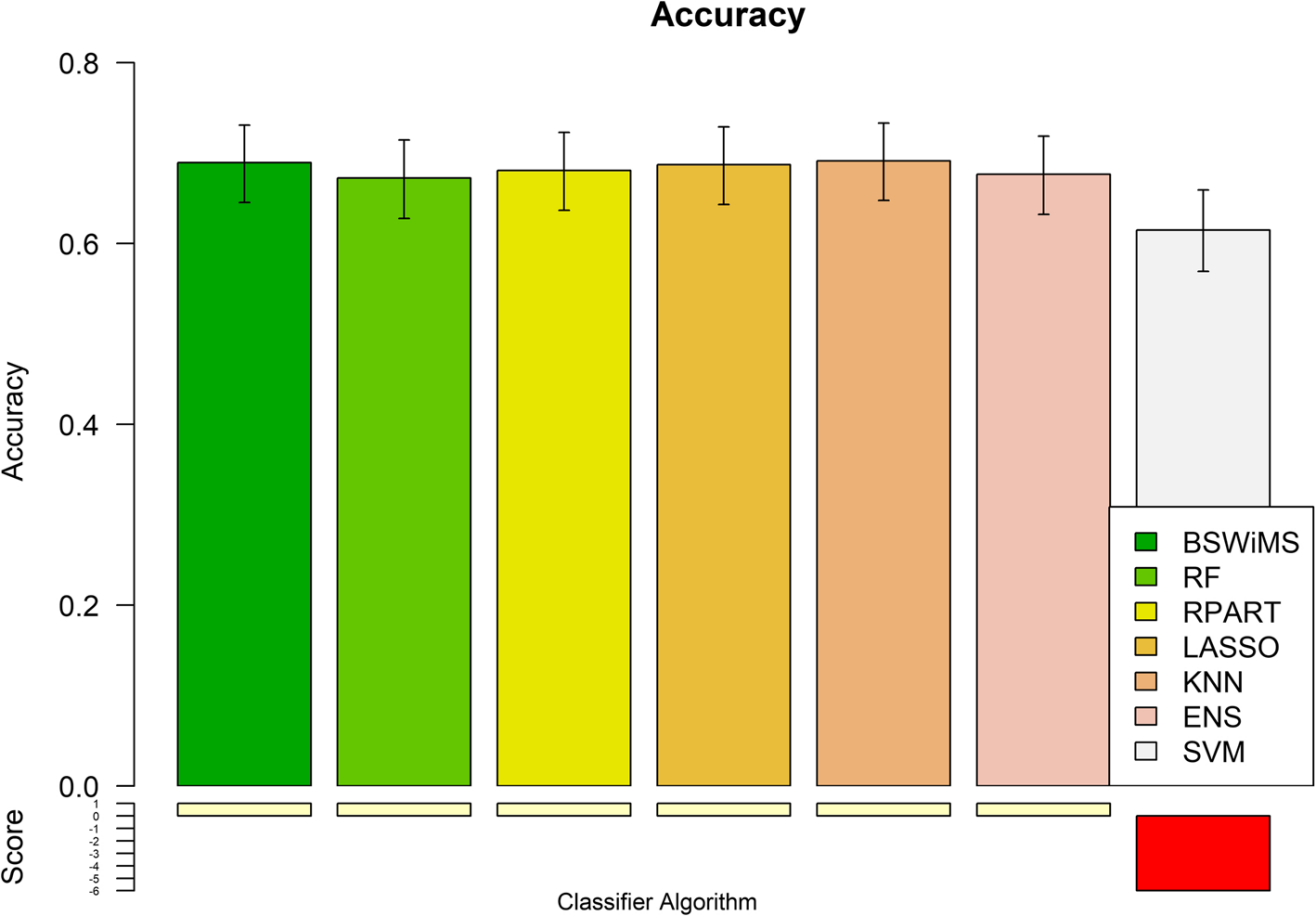
Accuracy of the FRESA.CAD Benchmark classifiers Comparison of the Accuracy obtained using the different classification methods of the FRESA.CAD Benchmarking with the ADNI-Discovery dataset for the Cross-validation and using the top 2500 SNPs as input

**Fig. 5:**
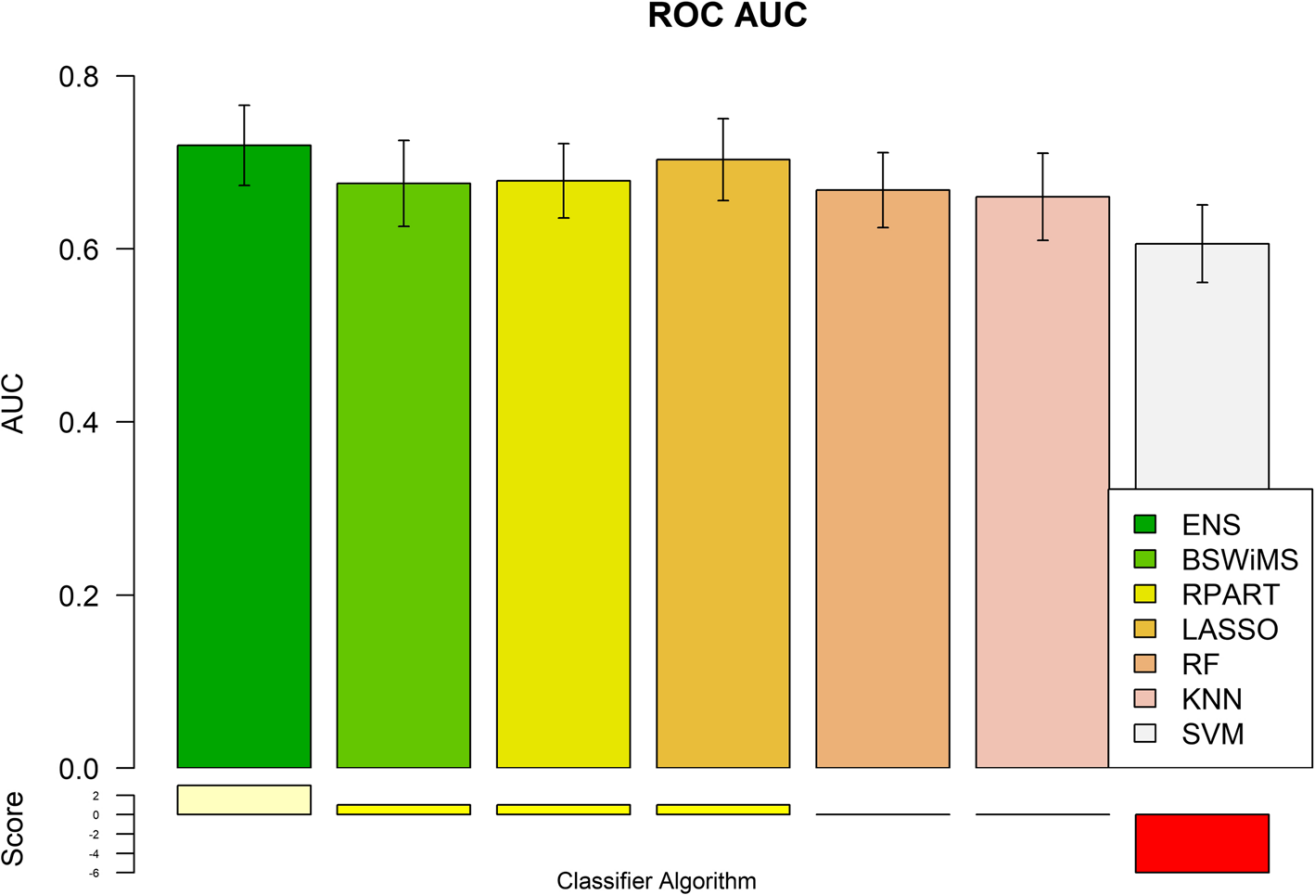
ROC AUC of the FRESA.CAD Benchmark classifiers Comparison of the ROC AUC Score obtained using the different classification methods of the FRESA.CAD Benchmarking with the ADNI-Discovery dataset for the Cross-validation and using the top 2500 SNPs as input

**Fig. 6:**
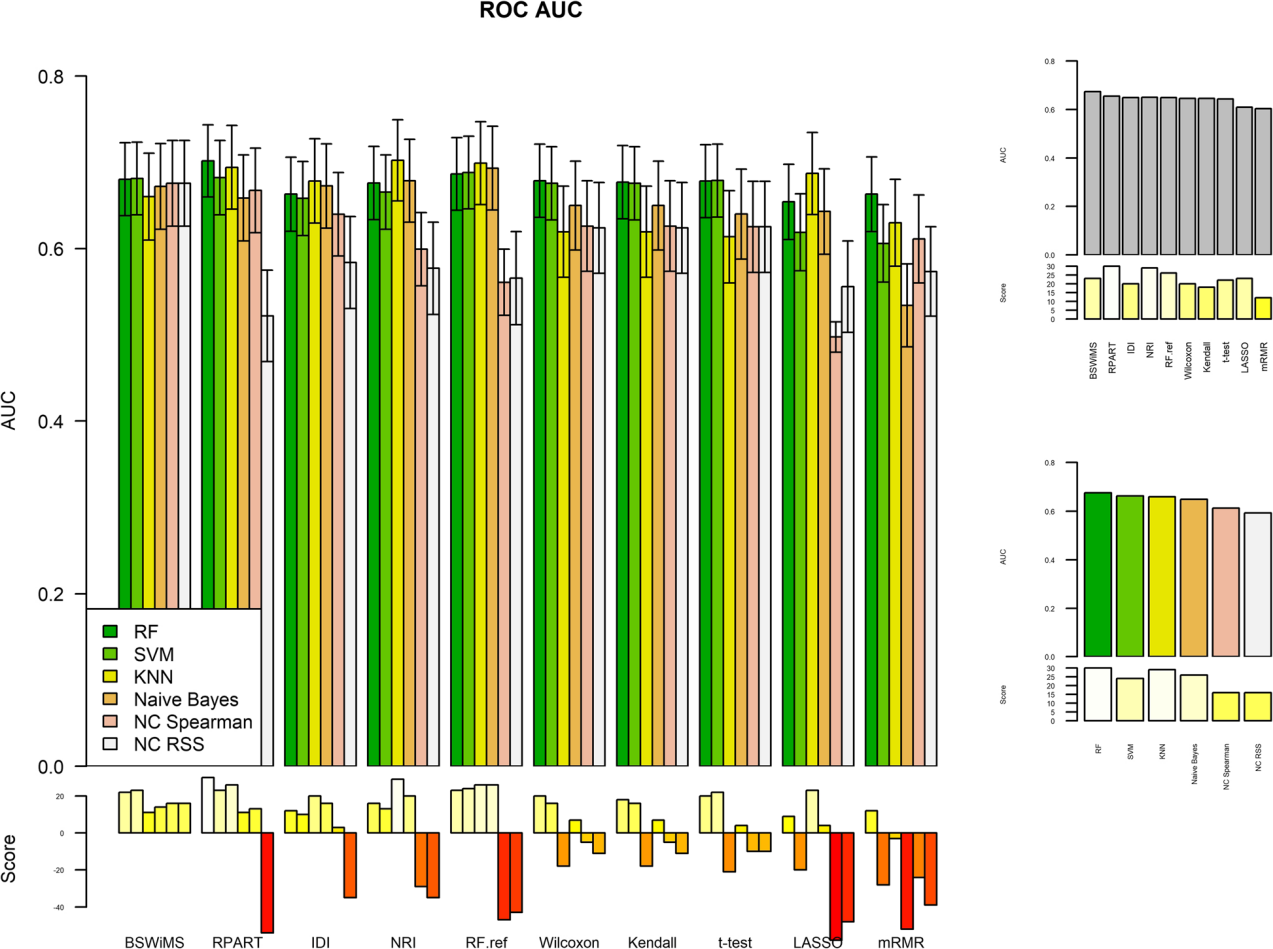
ROC AUC of the FRESA.CAD Filter combinations Comparison of the ROC AUC Score obtained using the different combinations of classification methods plus filters of the FRESA.CAD Benchmarking with the ADNI-Discovery dataset for the Cross-validation and using the top 2500 SNPs as input

**Fig. 7:**
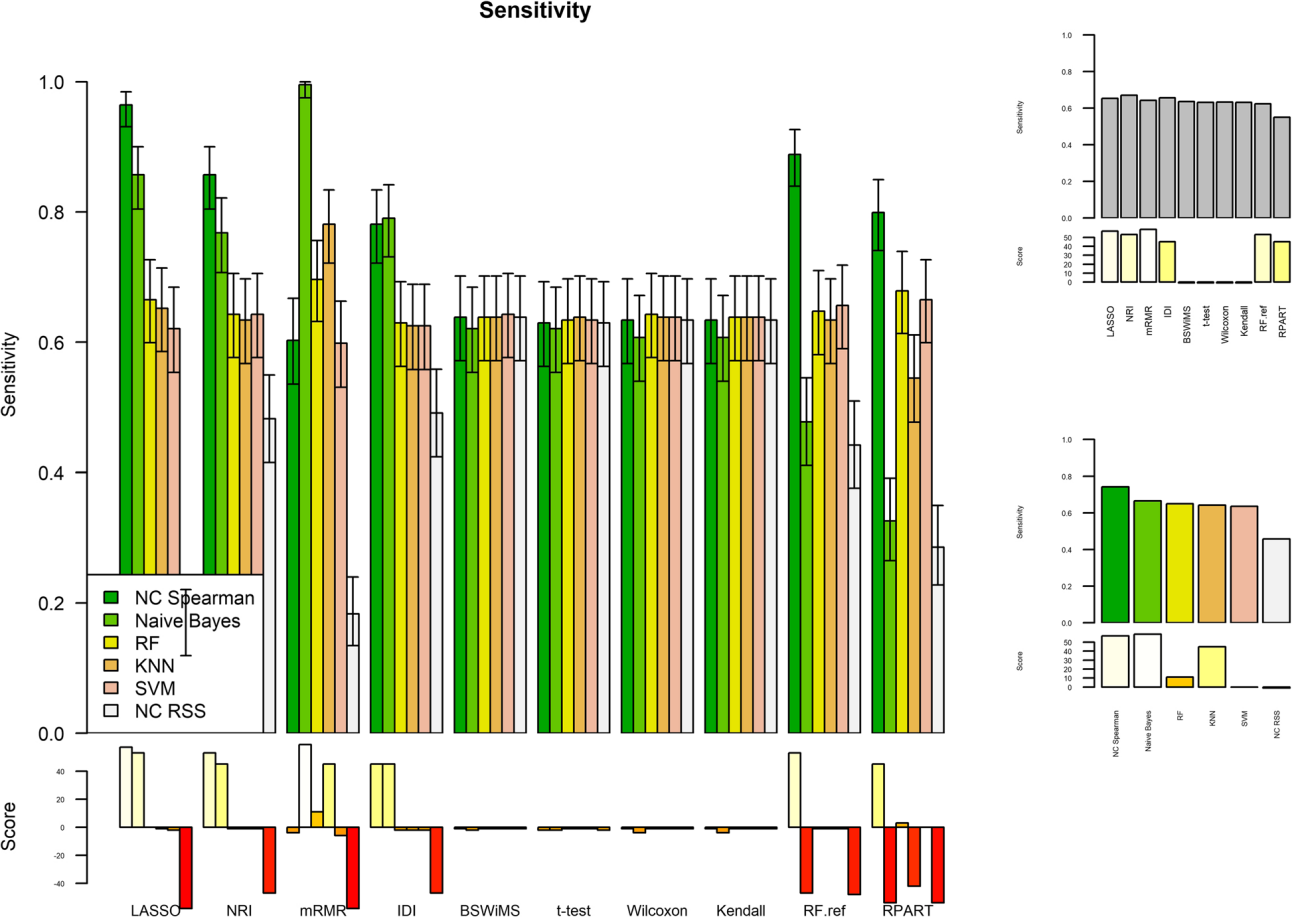
Sensitivity of the FRESA.CAD Filter combinations Comparison of the Sensitivity Score obtained using the different combinations of classification methods plus filters of the FRESA.CAD Benchmarking with the ADNI-Discovery dataset for the Cross-validation and using the top 2500 SNPs as input

**Fig. 8:**
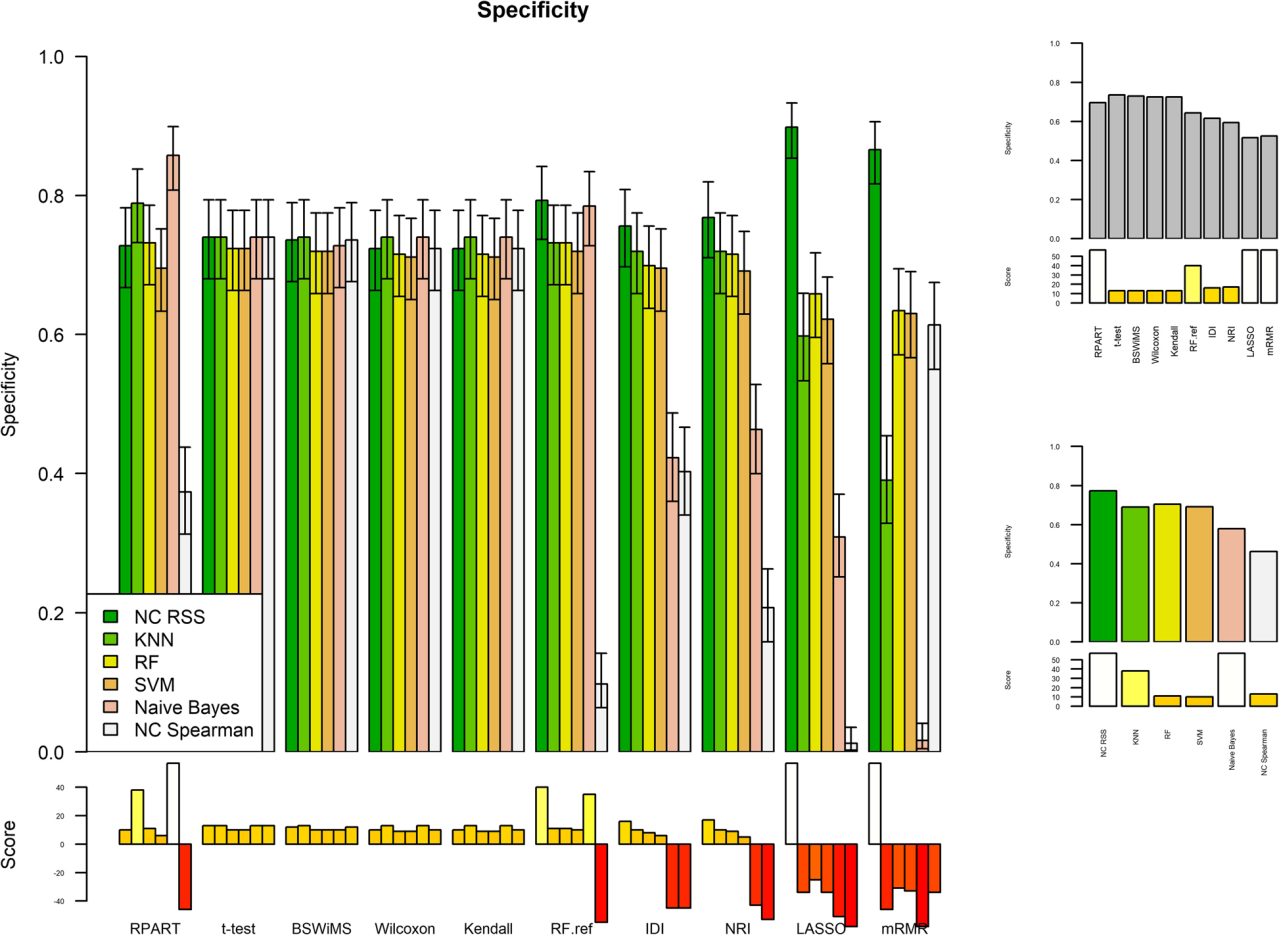
Specificity of the FRESA.CAD Filter combinations Comparison of the Specificity Score obtained using the different combinations of classification methods plus filters of the FRESA.CAD Benchmarking with the ADNI-Discovery dataset for the Cross-validation and using the top 2500 SNPs as input

Figures 1 and 2 show the Receiver Operating Characteristic Area Under the Curve (ROC AUC) of the ML methods on the ADNI dataset. The ROC AUC ranged from 0.60 to 0.70. The BSWiMS, LASSO, and RPART had equivalent performance, and the ensemble of the methods had the best performance with a ROC score of 0.719. Figures 3, 4, 5, 6, 7 and 8 show the detailed performance analysis of the ML methods. The balanced error, the ROC AUC, the accuracy as well as specificity and sensitivity for both classifiers and the combinations with filters are depicted as bar plots. These plots indicate that the support vector machine (SVM) engine with minimum redundancy maximum relevance (mRMR) filter had the lowest performance. On the other hand, the Least Absolute Shrinkage and Selection Operator (LASSO) method gave the best results among ML methods, which was further improved by using the Ensemble of methods and achieving a ROC AUC of 0.719.

**Fig. 9:**
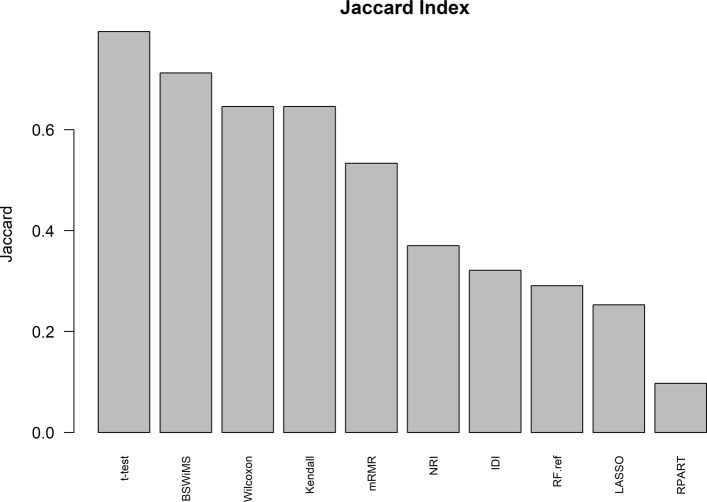
Jaccard Index Jaccard Index metric of the different classifiers between features selected by each classifier of the FRESA.CAD Benchmarking with the ADNI-Discovery dataset for the Cross-validation and using the top 2500 SNPs as input

**Fig. 10:**
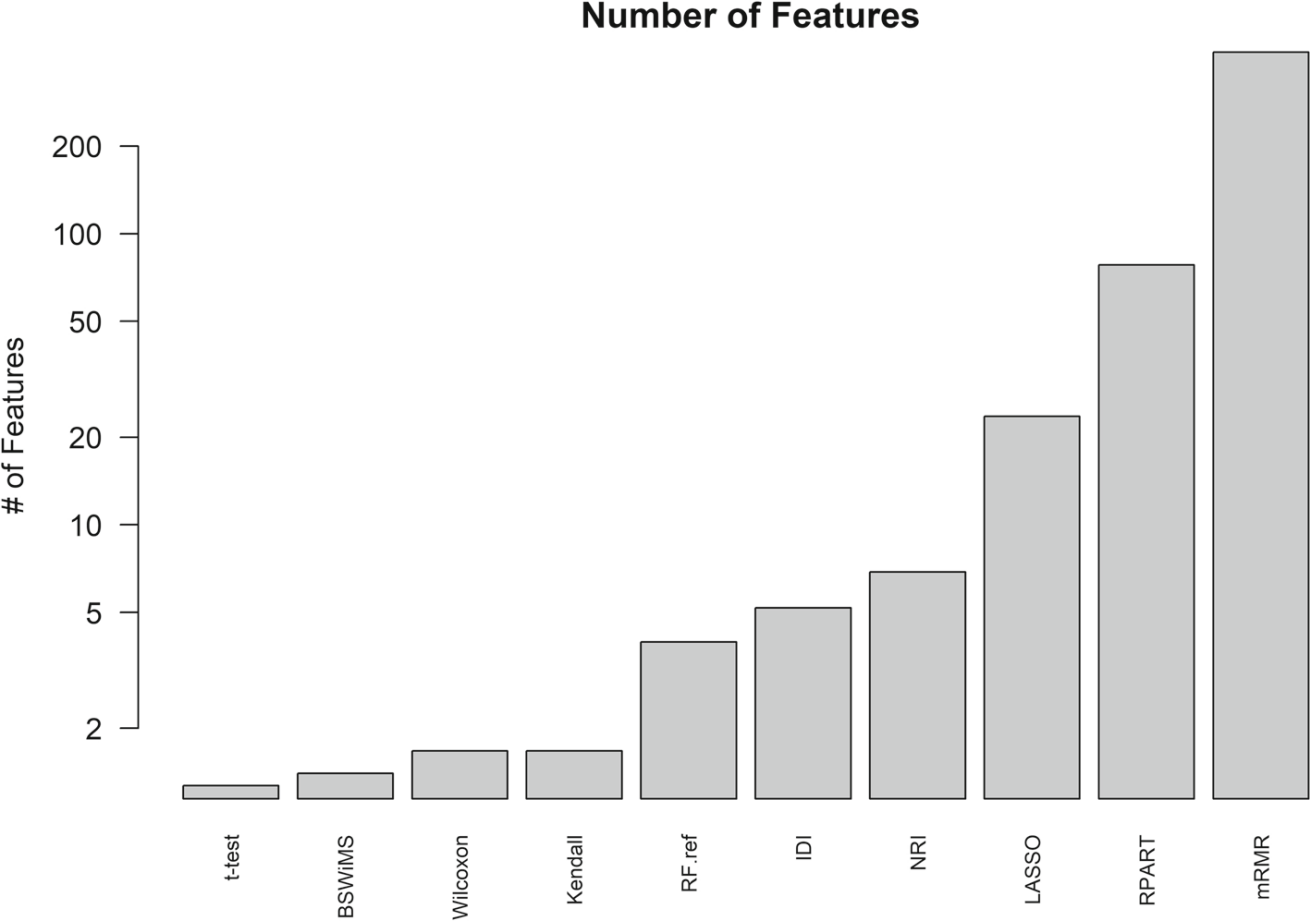
Number of Features The number of features selected by each classifier of the FRESA.CAD Benchmarking with the ADNI-Discovery dataset for the Cross-validation and using the top 2500 SNPs as input

**Fig. 11:**
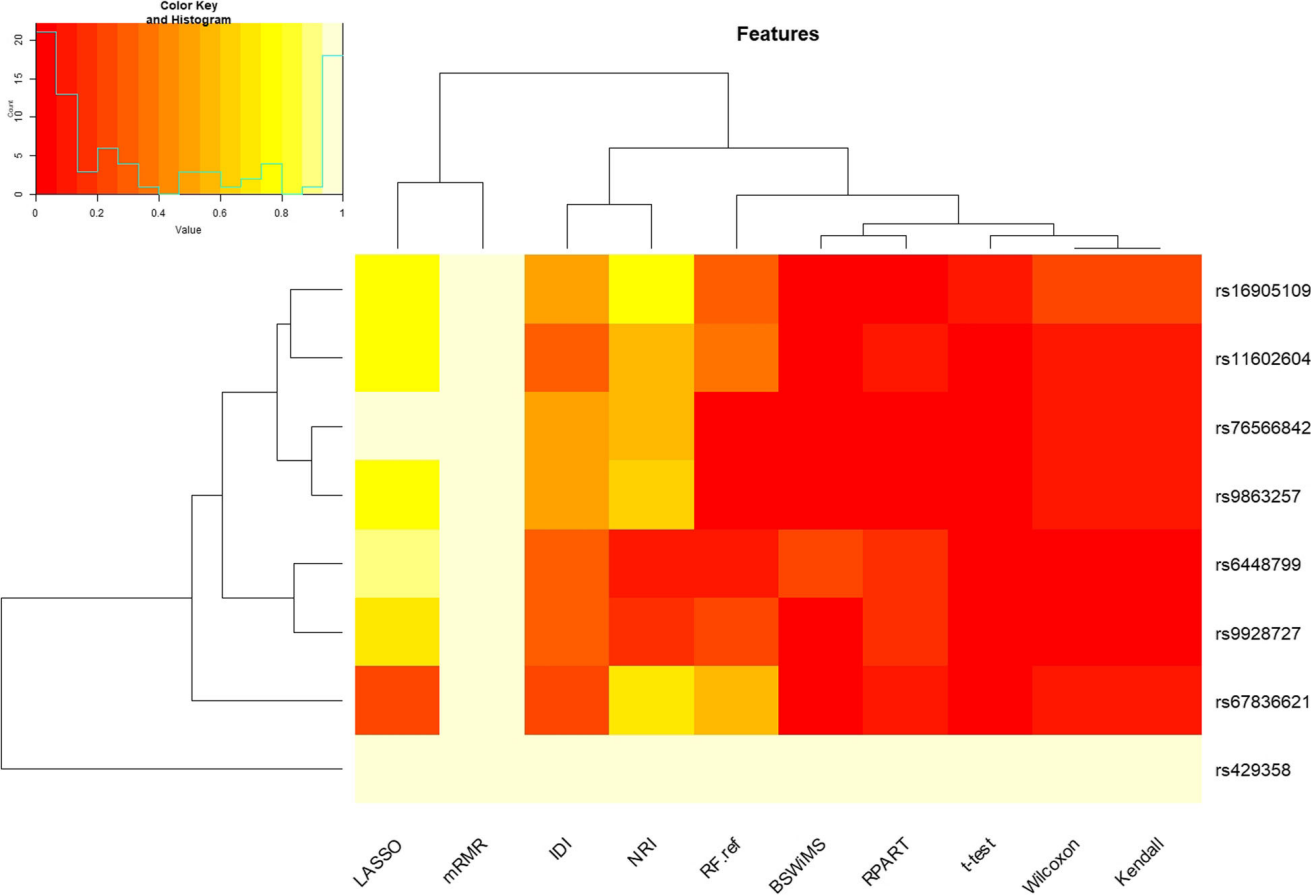
SNPs chosen more than 10% of the time as features of the FRESA.CAD Benchmark Heatmap of the main SNPs being chosen across all the classifiers. The Y axis are the main SNPs being selected while the X axis represents the different classifiers of the FRESA.CAD Benchmarking with the ADNI-Discovery dataset for the Cross-validation and using the top 2500 SNPs as input

Regarding feature selection: Fig. 9 shows the Jaccard index of the different methods, while Fig. 10 shows the average number of selected features. Finally, Fig. 11 shows the top selected features by the ML method and their selection frequency. These figures show that multivariate ML methods selected different features to construct their predictive models and that those features were not constantly selected at each one of the cross-validation repetitions. The method that constantly selected the same features was BSWiMS, but it was, on average, based on a single feature. On the other extreme, the mRMR filter selected on average over 200 features at each interaction; and 50% of the selected features were common between selection sets.

A detailed analysis of the results presented in Fig. 11 indicates that APOE *ε*4 (rs429358) was chosen by all the feature selection methods. LASSO is consistently using more SNPs than net reclassification improvement (NRI) filter and NRI selected more than the other filter methods. On the other hand, the classic mRMR filter selects many markers, but the cross validation (CV) performance results were not the best. The selection frequency analysis reported by the benchmark function shows that rs67636621, rs76566842, and rs16905109 deserve further exploration. Table 1 presents the results of the eight most important SNPs that were consistently selected by the ML methods (more than 10% across feature selection methods). Most of them had a significant association with the presence of AD according to the univariate Wilcoxon test (*p* <0.05). The APOE *ε*4 variant gives a very strong predictive power, and the remaining variants are then used to further improve the models. Table 1 also shows the location and the related genes of the top SNPs. One of the notable results is SNP rs6448799 which is a variant of LOC107986178 of the HS3ST1 gene. This gene has been shown to have a near study-wide association with the “backward digits” working memory, supporting association of these variants with AD and Mild Cognitive Disorder (MCI) [24].

**Table 1:** Characteristics of the top SNPs being selected as important features for the ADNI-Discovery Dataset

SNP	Location	Function	Gene	Gene summary	WILCOX	FREQ
rs429358	19:44908684	Missense Variant	APOE	APOE is a protein coding gene which generates alipoprotein E, a fat-binding protein crucial in many mechanisms of the body. This gene is related to Alzheimer’s Disease and Lipopoprotein Glomerulopathy among others.	0	1.000
rs67836621	19:51186703	Noncoding (Intergenic)	Adjacent: SIGLEC20P, LOC100133225 (Pseudogene)	Unknown	8e-04	0.298
rs9928727	16:9018042	Noncoding (Intergenic)	Adjacent: LOC105371074 (Uncharacterized), C16orf72	Unknown	9e-04	0.269
rs11602604	11:62231065	Noncoding (Intergenic)	Adjacent: SCGB2A1, SCGB1D2	Unknown	3e-04	0.321
rs6448799	4:11628425	Intron Variant	HS3ST1 (LOC107986178)	HS3ST1 is a protein coding gene which is crucial to create heparan sulfate structures that participate in sulfotransferase activity. This gene is related to Arteriosclerosis and Coronary Heart Disease.	6e-04	0.288
rs16905109	8:134194872	Noncoding (Intergenic)	Adjacent: LOC100419617 (Pseudogene), ZFAT	Unknown	0.0011	0.383
rs76566842	9:28296478	Intron Variant	LINGO2	LINGO2 is a protein coding gene for the Leicine-rich Repeat Neuronal Protein. This gene is related to the Essential Tremor disease.	0.1619	0.327
rs9863257	3:27586911	Noncoding (Intergenic)	Adjacent: RNU1-96P, RPS27P11	Unknown	0.1955	0.323

**Fig. 12:**
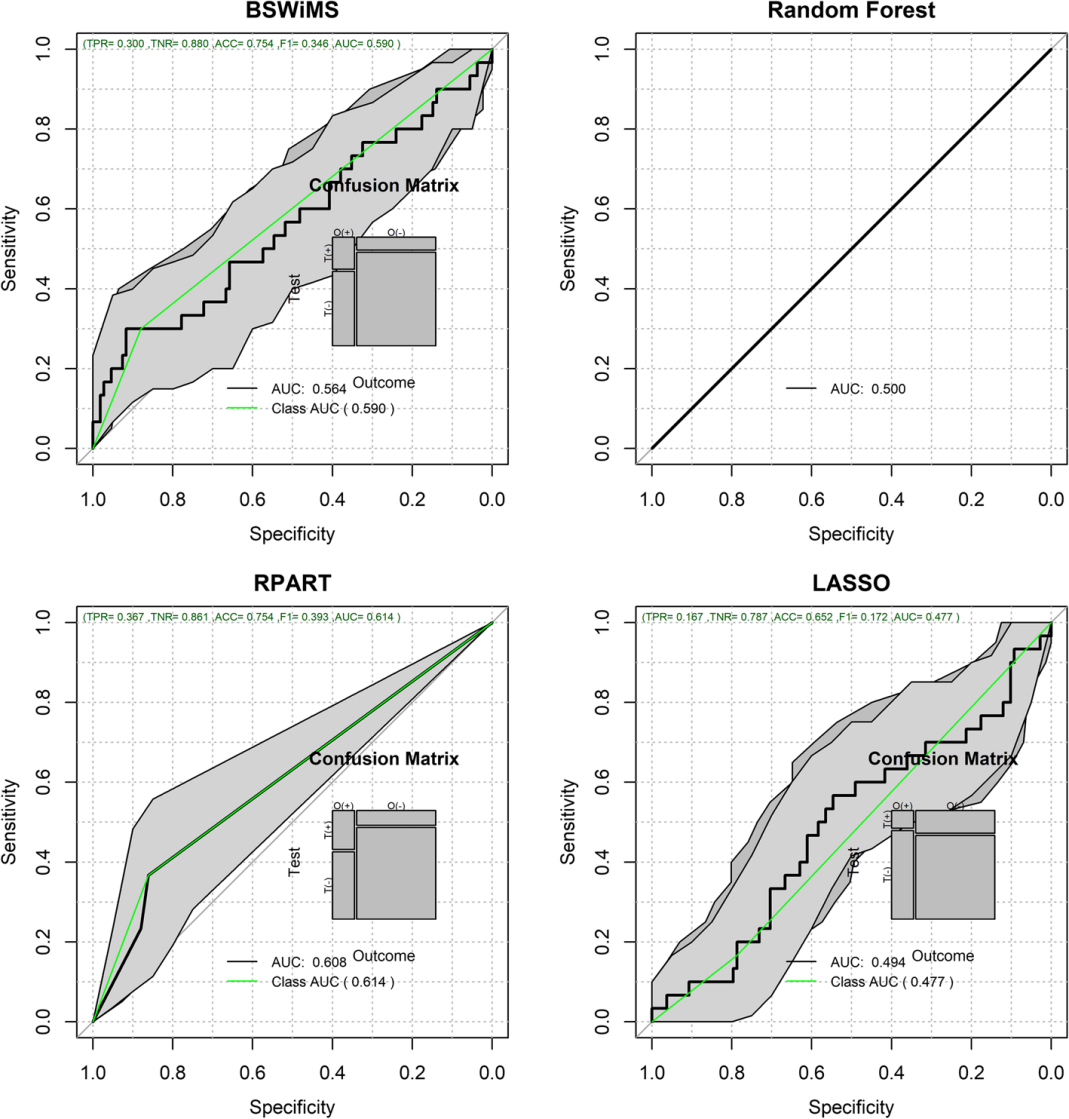
Validation ROC Curves for the FRESA.CAD Benchmarking Classifiers ROC Curves obtained using BSWiMS, Random Forest, RPART and LASSO of the FRESA.CAD Benchmarking with the ADNI-Validation dataset for the Cross-validation and using the top 1000 SNPs as input

**Fig. 13:**
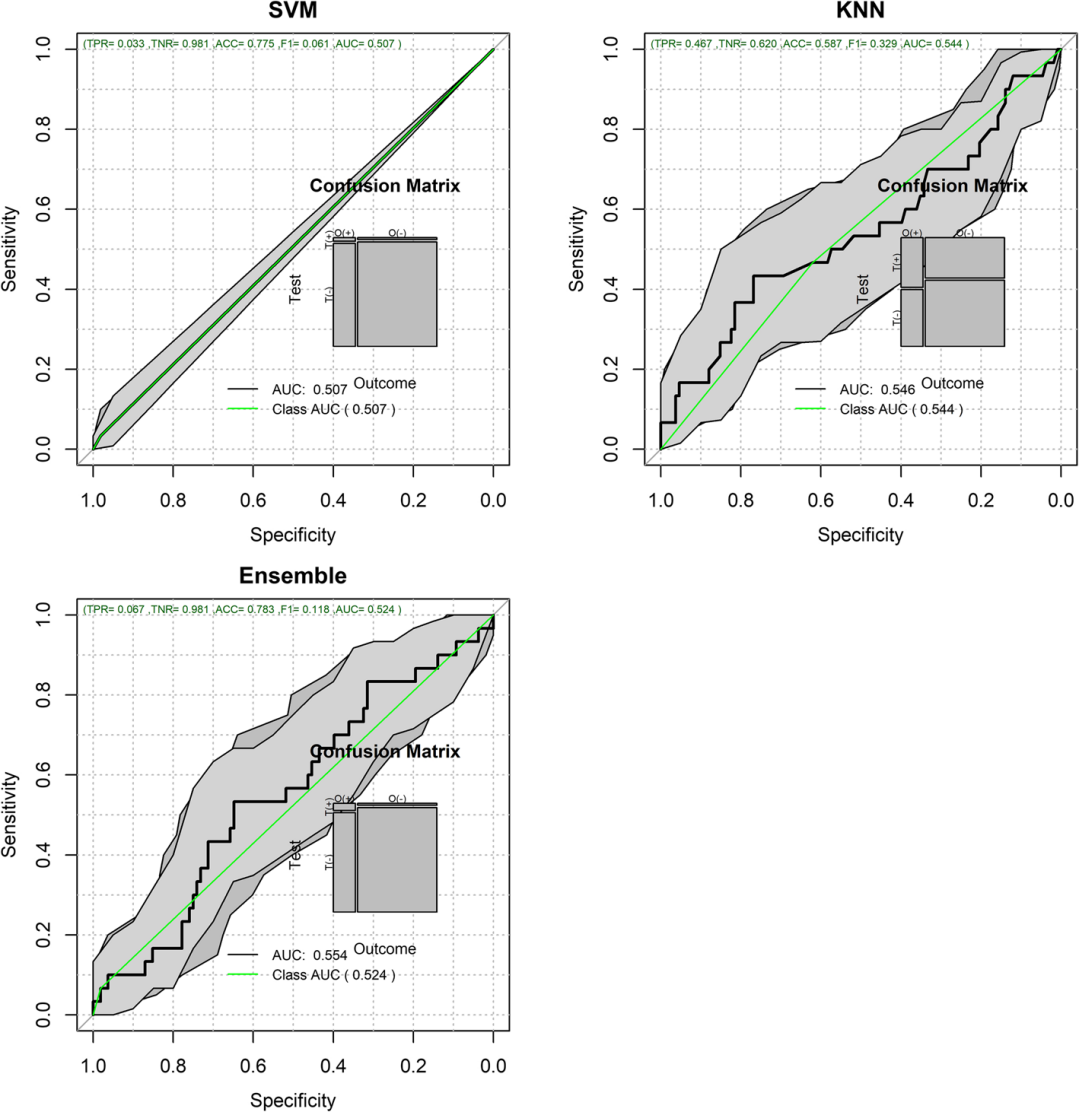
Validation ROC Curves for the FRESA.CAD Benchmarking Classifiers (Continued) ROC Curves obtained using SVM, KNN and the Ensemble of the FRESA.CAD Benchmarking with the ADNI-Validation dataset for the Cross-validation and using the top 1000 SNPs as inputs

Figures 12 and 13 show the validation performance results of the benchmarked ML methods based on the top 1000 SNP obtained from the IGAP-independent data set. The ROC AUC ranged from 0.50 to 0.65, and the balanced error rate (BER) ranged from 0.5 to 0.39. Filtered Naive Bayes (AUC= 0.65, BER=0.42) was the top ML method, followed by RPART (AUC=0.63, BER=0.39).

**Fig. 14:**
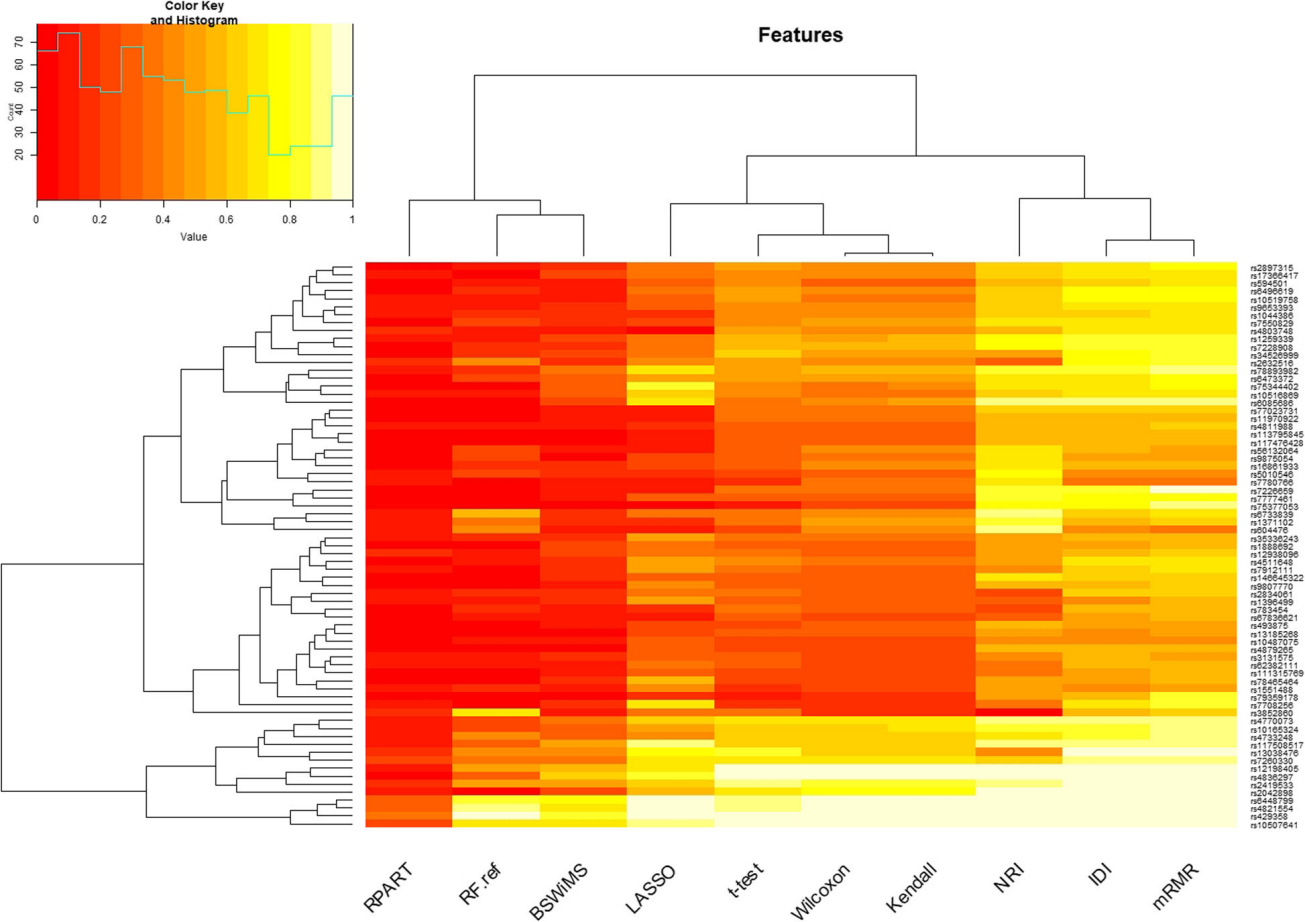
Validation SNPs chosen more than 10% of the time as features of the FRESA.CAD Benchmark Heatmap of the main SNPs being chosen across all the classifiers. The Y axis are the main SNPs being selected while the X axis represents the different classifiers of the FRESA.CAD Benchmarking with the ADNI-Validation dataset for the Cross-validation and using the top 1000 SNPs as input

The feature selection analysis of the validation returned a larger set of SNPs candidates. Figure 14 and Table 2 show the set of SNPs that were selected at least 10% of the time. Despite the large number of SNPs only APOE *ε*4 and rs6448799 appeared on both the full ADNI and IGAP-independent validation set.

**Table 2:** Characteristics of the top 10 SNPs being selected as important features for the ADNI-Validation Dataset

SNP	Location	Function	Gene	Gene summary	WILCOX	FREQ
rs429358	19:44908684	Missense Variant	APOE	APOE is a protein coding gene which generates alipoprotein E, a fat-binding protein crucial in many mechanisms of the body. This gene is related to Alzheimer’s Disease and Lipoprotein Glomerulopathy among others.	0	1.000
rs6448799	4:11628425	Intron Variant	HS3ST1 / LOC107986178	HS3ST1 is a protein coding gene which is crucial to create heparan sulfate structures that participate in sulfotransferase activity. This gene is related to Arteriosclerosis and Coronary Heart Disease.	6e-04	0.288
rs4821554	22:36880042	Noncoding (Intergenic)	Adjacent: NCF4, LOC105373022 (Uncharacterized)	Unknown	1e-04	0.874
rs7260330	19:44932959	Noncoding (Intergenic)	Adjacent: APOC1P1, APOC4-APOC2	Unknown	0.0027	0.667
rs10507641	13:59857910	Intron Variant	DIAPH3,	DIAPH3 is a protein coding gene that generates a Diaphanous forming protein, which regulates cell movement and adhesion. It is related to Auditory Neuropathy and Neuropathy	0	0.797
rs4733248	8:31302383	Intron Variant	LOC101929492 (Uncharacterized)	Unknown	0.0052	0.581
rs13038476	20:4158146	Intron Variant	SMOX	SMOX is a protein coding gene that generates the Spermine Oxidase which helps as neurotransmitters and cell regulators. It is related to Short-Rib Thoracic Dysplasia and Acute Hemorrhagic Leukoencephalitis	0	0.627
rs2419533	4:132668359	Intron Variant	LINC01256	LINC01256 is a non-coding RNA gene	0.0013	0.716
rs34526999	5:33728435	Intron Variant	ADAMTS12	ADAMTS12 is a protein coding gene that generates ADAMTS which works in pulmonary cell development or tumor processes. It is related to Brachydactyly and Intrahepatic Cholestasis of Pregnancy	0.025	0.445
rs2632516	17:58331728	Intron Variant	TSPOAP1-AS1	TSPOAP1-AS1 is a non-coding RNA gene	0.02	0.387

## Discussion

Most of the experimental treatments in development for LOAD require implementation at the very early stages of the disease to be effective [25]. Genetic approaches to predicting the risk of LOAD are a powerful and viable alternative to traditional biomarker-based disease prediction methods [26]. Traditional GWAS have only found SNPs that so far can only explain 33% of the estimated 79% [8] fraction of genetic risk associated with Alzheimer’s disease. While this value is low for a reliable clinical prediction, Machine learning methods have been proven to perform better in detecting candidate SNPs and predicting complex genetic diseases such as Type-2 Diabetes [27], Inflammatory Bowel Syndrome [28] and Obesity [29]. The use of machine learning-based approaches for Genetic-based Precision Medicine has increased in the current decade and shows signs of increasing [30].

This study presented the hypothesis that Benchmarking ML methods on SNP dataset can aid in discovering novel SNPs associated with the late onset of AD. Specifically, we studied the capability of the FRESA.CAD benchmarking method to discover and model the genetic risk factor. Benchmarking allowed us to gain insight in the degree of genetic risk associated with LOAD by comparing and analyzing multiple Machine Learning models applied to predict the risk a person of developing Alzheimer’s Disease from genetic information only. The Machine Learning models were expected to find linear and nonlinear relationships between genes that could explain more of the missing heritability of Alzheimer’s disease. Constructing models with the capability to detect epistasic relationships would be an important advancement compared to traditional single-variant GWAS studies. The results show that some models obtained promising results in predicting the development of the disease, namely BSWiMS, LASSO, RPART, and the Ensemble. The best ROC AUC score achieved with the ADNI-Discovery was ∼0.719 and 0.61 in the IGAP-independent subset. This result is promising considering the upper boundary set by the calculated heritability from purely genetic components (79% as described in [8]). Furthermore, the model outperforms those methods which only use the APOE4 gene, which achieve around 63 65%, and simple deep learning models, which achieve 62%.. It is noteworthy that this study showed marked differences between the ML methods in modeling LOAD. On the other hand, the ADNI results indicated a small subset of SNPs that can be used in multivariate models, while the independent IGAP study returned hundreds of possible candidates.

The models tested with the FRESA.CAD Benchmark indicated that the ensemble method had a sensitivity of 70% with a specificity of 65%, implying a strong genetic risk component in the ADNI cohort.. We also found that different feature selection methods selected common SNPs that have been already associated with Alzheimer. Thus, SNP selection based on set overlap may be a powerful method to discover clinically significant risk factors. The reduced cohort for the confirmatory validation indicated that the Naive Bayes classifier had a sensitivity of 33% with a strong specificity of 95%. The contradictory findings between the full dataset and the validation subset may be a class imbalance problem coupled with limitations regarding the size of the dataset. Regardless of the differences between cohorts, the presented results support the previous SNP finding that the APOE *ε*4 gene is the main risk factor for Late Onset Alzheimer’s disease [31]. Furthermore, we were able to confirm a new possible variant associated with the disease: rs6448799. According to recent GWAS studies, this last genetic variant may have a true correlation with Alzheimer’s Disease [24, 32]. Hence, FRESA.CAD Benchmark seems to be a promising tool for Genomics analysis and finding candidate clinical markers. This study is limited by the small sample size; we expect that the predictive capability of the machine learning models can be improved by increasing the sample size. Therefore, we believe that these models hold much promise for the clinical diagnosis of Late-Onset Alzheimer’s Disease and other complex diseases.

The upper limit of the genetic component alone presents a challenge for the highly precise accuracy required for a clinical diagnostic. One of the possible solutions for this problem would be to complement the genetic-based methods with imaging or clinical data. The genetic analysis could be used to detect those individuals with a higher risk of developing Alzheimer’s Disease, and then those individuals could be monitored on a yearly basis with imaging technologies to detect the development of the disease at the earliest possible moment.

LOAD polygenic scores currently available are not capable to predict mild cognitive impairment to LOAD progression [33]. Therefore, alternative models are also required for the accurate prediction of disease progression. Additionally, alternative hypothesis such as Pritchard’s Omnigenetics [34] could also be explored efficiently using ML methods to model and identify cellular networks and the respective flow of regulatory information, finding a more comprehensive and general solution.

## Conclusions

This research study has shown the results of applying the FRESA.CAD Binary Classification Benchmarking algorithms to predict the risk of developing Late-Onset Alzheimer’s Disease from genetic variation data exclusively. Conducting systematic comparisons on the classification performance of machine learning algorithms is a crucial task for achieving the predictive potential of these models. Model selection methodologies used to optimize machine learning models also hold the potential for the discovery of new genetic markers associated with the disease. Given that the preliminary results show promise, we believe that a refined model could be a powerful tool for the prediction and early detection of this disease. The current models show limitations due to the complexity of the disease and the size of the datasets, both of which stand to benefit from the increasing availability of data. This paper also demonstrates that Machine Learning methods are powerful tools suited to analyze and leverage a multitude of genes that could be used in a variety of complex diseases similar to Alzheimer’s Disease. The current technological trend points toward the large-scale application of these methods with the ever-increasing demand for individual genome sequencing and the availability of much larger datasets.

## Methods

Data used in the preparation of this article were obtained from the Alzheimer’s Disease Neuroimaging Initiative (ADNI) database (http://adni.loni.usc.edu). The ADNI was launched in 2003 as a public-private partnership, led by Principal Investigator Michael W. Weiner, MD. The primary goal of ADNI has been to test whether serial MRI, PET, other biological markers, and clinical and neuropsychological assessment can be combined to measure the progression of MCI and early AD.

We selected individuals who have either a Cognitively Normal or Alzheimer’s Disease. PLINK [19, 20] was used to read the Variant Call Format data of the WGS and to convert it to the more compact format of Binary Pedigree Files (BED). After that, we used Python 3.5 and the library PyPlink [21] to perform quality control procedures in a similar pipeline to the one described by Turner [22].

We began by performing pre-quality controls on the samples, using marker call rate, sample call rates and Minor allele frequency (MAF) filtering. Once this is done Identity-By-Descent (IBD) is performed with a value of 0.25 to find those individuals related to each other to be removed. After the binary classification filter and the IBD filter the samples are reduced from 808 individuals to 471 individuals. We named this the ADNI-Discovery dataset, it is balanced in terms of cases/controls, has a mean age of 75.5 and it is slightly skewed towards males, as is shown in Table 3.

**Table 3:** Dataset and validation subset demographic metrics

Dataset	Size	Male	Female	Mean age	Controls	Alzheimer’s cases
ADNI-Discovery	471	252	219	75.57	241	230
ADNI-Validation	167	92	75	72.17	130	37

Afterwards, marker call rate (≤99*%*) and MAF filtering (≤0.01) are used to reduce the number of SNPs to only those that are useful. Then, the Hardy-Weinberg Equilibrium test is done (≤0.05) to further clean SNPs. Finally LD-Based clumping (*p*-value ≤0.01, *r*^2^≤0.05) is used to find those SNPs which are in Linkage Equilibrium and are statistically relevant. For a correct LD-based clumping the statistical data used as reference should be obtained from a different data set which is sufficiently large. In our case we used the statistical summary results from the International Genomics of Alzheimer’s Project (IGAP) [23] to guide the clumping algorithm and find the statistically relevant and independent candidate SNPs. These summary statistics are generated from 74,046 individuals. The Quality Control Pipeline returned 8,239 SNPs in Linkage Equilibrium after performing the LD-clump based on the IGAP Summary Statistics. Finally, for performance reasons, we reduced these 8,239 SNPs to only the top 2,500 SNPs based on their *p*-value (ascending) as an input to the benchmarking tool. The ADNI dataset was selected as the base of the analysis even though it has a much smaller sample size as it has the full WGS data available for each subject, while the IGAP only makes the summary statistics openly available.

For further validation, we also generated a second validation subset from the dataset where we took only those individuals in the ADNI which did not take part in the IGAP study for validation as there were some existing individuals present in both datasets. Due to the reduced data set size we further reduced the SNPs used as input to just the top 1,000 SNPs (Also based on their ascending *p*-value). In contrast with the full dataset, the validation set is highly unbalanced, with 78% of the samples being controls, the mean age is slightly lower as shown in Table 3.

Multivariate model-building and validation were done using the FRESA.CAD Benchmarking tool that runs the following ML methods:

- Bootstrap Stage-Wise Model Selection (BSWiMS), or user-supplied cross-validated (CV) method.
- Least Absolute Shrinkage and Selection Operator (LASSO)
- Random Forest (RF)
- Recursive Partitioning and Regression Trees (RPART)
- K Nearest Neighbors (KNN) with BSWiMS features
- Support Vector Machine (SVM) with minimum-Redundancy-Maximum-Relevance (mRMR) feature selection filter
- The ensemble of all the above methods

The CV performance of these classification algorithms is also complemented with the following feature selection algorithms and different filters: BSWiMS, LASSO, RPART, RF, integrated discrimination improvement (IDI), net reclassification improvement (NRI), t student test, Wilcoxon test, Kendall correlation, and mRMR as filters on the following classifiers: KNN, naive Bayes, nearest centroid (NC) with normalized root sum square distance and Spearman correlation distance, RF and SVM.

The results of the CV instances executed by the binary benchmark were compared using the performance statistics and ranked by their 95% confidence interval (CI). The ranking method accumulates a positive score each time the lower CI of a performance metric is superior to the mean of the other methods and loses a point each time the mean is inferior to the top 95% CI of the other methods. The package returns the accuracy, precision, sensitivity, the balanced error rate and the ROC AUC with their corresponding 95% confidence intervals (95% CI). We used the ranking results to infer the suitability of ML methods to predict AD in the ADNI dataset.

Finally, we independently analyzed the validation subset (IGAP-independent) using the FRESA.CAD benchmarking procedure.

## Data Availability

The datasets generated and/or analyzed during the current study are available in the ADNI LONI repository, http://adni.loni.usc.edu/

## Abbreviations

AD: Alzheimer disease
ADNI: Alzheimer’s disease neuroimaging initiative
APOE: Apolipoprotein E
BED: Binary pedigree files
BER: Balanced error rate
BSWiMS: Bootstrap stage-wise model selection
CI: Confidence interval
CV: Cross validation
EOAD: Early-onset alzheimer’s disease
FRESA.CAD: Feature selection algorithms for computer aided diagnosis
GWAS: Genome-wide association studies
IBD: Identity by descent
IDI: Integrated discrimination improvement
IGAP: International genomics of alzheimer’s project
KNN: K nearest neighbours
LASSO: Least absolute shrinkage and selection operator
LOAD: Late-onset alzheimer’s disease
MAF: Minor alelle frequency
MCI: Mild cognitive impairment
ML: Machine learning
MRI: Magnetic resonance imaging
mRMR: Minimum redundancy maximum relevance
NC: Nearest centroid
NRI: Net reclassification improvement
PET: Positron emission tomography
RF: Random forest
ROC: AUC Receiver operating characteristic area under the curve
RPART: Recursive partitioning and regression trees
SNP: Single nucleotide polymorphism
SVM: Support vector machine
